# An experimental look at trust, bargaining, and public goods in fishing communities

**DOI:** 10.1038/s41598-021-00145-5

**Published:** 2021-10-21

**Authors:** Cristian A. Rojas, Joshua Cinner, Jacqueline Lau, Cristina Ruano-Chamorro, Francisco J. Contreras-Drey, Stefan Gelcich

**Affiliations:** 1 Instituto Milenio en Socio-Ecología Costera and Center of Applied Ecology and Sustainability (CAPES), Departamento de Ecología, Pontificia Universidad Católica de Chile, Santiago, Chile; 2 ARC Centre of Excellence for Coral Reef Studies, James Cook University, Townsville, QLD 4811 Australia; 3 WorldFish, Batu Maung, Penang Malaysia

**Keywords:** Psychology and behaviour, Environmental social sciences, Environmental economics

## Abstract

Pro-social behavior is crucial to the sustainable governance of common-pool resources such as fisheries. Here, we investigate how key socioeconomic characteristics influence fishers’ pro-social and bargaining behavior in three types of experimental economic games (public goods, trust, and trade) conducted in fishing associations in Chile. Our games revealed high levels of cooperation in the public goods game, a high degree of trust, and that sellers rather than buyers had more bargaining power, yet these results were strongly influenced by participants’ socioeconomic characteristics. Specifically, gender, having a secondary income source, age, and being the main income provider for the household all had a relationship to multiple game outcomes. Our results highlight that engagement in pro-social behaviors such as trust and cooperation can be influenced by people’s socioeconomic context.

## Introduction

Small-scale fisheries provide employment and nutrition for millions of people^1^. Yet the continued support of people’s livelihoods and wellbeing requires fisheries to be sustainably governed^1,2^. Sustainable governance of fisheries often requires behaviors such as cooperation, trust, and reciprocity. More specifically, many small-scale fisheries are managed as common property regimes^3^, relying on pro-social behavior for enforcing local rules, solving conflicts, transferring knowledge, and ensuring sustainable extraction^4^. Along with pro-social behaviors, bargaining can be essential for small-scale fisheries’ subsistence and equity. Many fisheries are connected to local and more distant markets, and small-scale fisheries’ livelihoods depend on fish being productively bartered or sold as a source of income^5^. Bargaining power can influence how fishers benefit from markets. Those with less power may have less ability to benefit. For instance, women may have less bargaining power than men in negotiations, which can contribute to persisting gender inequities^6,7^. In addition, successful market transactions are connected to pro-social behaviors of trust and trustworthiness. As such, a greater understanding of cooperation, trust—hereafter pro-social behavior—and bargaining in fisheries provides useful insight into how people within small-scale fisheries might respond to changes in management and markets, which is critical for informed governance.

Increasingly, decision-makers and global organizations have emphasized the need for inclusive management and governance of small-scale fisheries. Within this mandate^8^, is the recognition that expectations, norms and behaviors within small-scale fisheries shape and are shaped by socio-cultural and socioeconomic characteristics, such as gender and class. Critically, identities—like gender—do not exist in a vacuum, nor function alone. Instead, other socioeconomic characteristics—such as age, marital status, class or ethnicity—coexist and interact to shape social norms, behavior, and outcomes^9,10^. Unfortunately, only a small number of studies examine how pro-social behavior in fisheries is related to a suite of socioeconomic characteristics. For instance, age could be associated with different time or risk preferences^11^; income with financial dependency on the resource; and marital status with community ties. Here, we seek to understand how these individual characteristics shape pro-social and bargaining behaviors in small-scale fishing communities.

People’s choices in simulated situations—games—can help understand pro-social behavior and bargaining behaviors. By creating incentive-compatible situations that isolate people’s actions, games allow us to understand behaviors that might be attributed to specific socioeconomic characteristics (e.g., gender) that might otherwise be difficult to observe, due to public image, peer pressure, self-reporting bias, culture, etc. Thus, games are particularly useful for understanding how social-economic characteristics might be implicated in people’s behavior. However, few studies have used games to examine multiple facets of behavior in fishing communities. Here, we use behavioral economics tools to explore how participant’s characteristics influence three types of behavior relevant to the governance of the commons: cooperation, trust, and bargaining.

Public goods games are used to understand cooperation and free riding behaviors, common features of common-pool resources like fisheries^12,13^. These games typically involve an experimental setting which allows people to contribute or not to a public good. In the game, the highest payoffs for an individual occur when everyone else contribute, and the lowest is when that individual contributes, but no one else does. Contributions to the public good can be used as a measure of cooperation. Not contributing anything is often seen as a measure of selfishness; individuals who do not contribute are ‘freeriders’ who benefit from the contributions of others while contributing nothing.

Trust games are used to understand levels of social cohesion, which can influence conflict resolution and enforcement in fishing communities that self-manage their resources^4^. Trust games typically involve an experimental setting whereby one player can send money to another player, which is multiplied by the experimenter (e.g., doubled or tripled). The recipient is then asked whether they want to send money back to the original sender. Thus, the payoffs for the first player are highest if they trust the second player and that trust is reciprocated. Money sent can be used as a proxy for the level of trust from senders while money sent back by receivers can be seen as a measure of trustworthiness (or reciprocity) for receivers.

Finally, trade or bargaining games can be used to understand how fishing communities managing commons will react to market forces, which are a major driver of change in small-scale fisheries^14,15^. Bargaining games involve an experimental setting where participants are assigned the role of buyers or sellers, provided an endowment (money for buyers and a tradable object for sellers), and tasked with reaching a trade agreement. Payoffs are structured such that if a bargaining agreement is reached, buyers get their endowment minus the agreed price and sellers get the price of trade. Offers to buy (bids) and sell (asks) as well as equilibrium prices can serve as reference for bargaining power, while earning from trade can be used as a measure of bargaining prowess.

Until recently, most behavioral economic studies have been carried out in developed countries using university students^16,17^. These studies likely misrepresented the behavior of people who are not formally educated, affluent students. While the trend has changed in the last decade, with several studies carried out in the field and in developing countries, studies in fishing communities are rare and often have been limited to public goods^12,13,18,19^; very few studies have examined trust and reciprocity games or bargaining games^20^. As such, further work in communities that rely on and are involved in managing common-pool resources is necessary.

We conducted three independent economic games (trust, bargaining, and public goods) with different participants, in an experimental setting on the field^21–23^, with small-scale fishing communities of the Valparaiso region in the central coast of Chile. Small-scale fishers in Chile target multiple demersal and benthic resources (e.g., hake, jumbo squid, kelp, shellfish) through diving, fishing and gleaning, are organized in fishing associations, and operate under different fishing management regimes (e.g., harvest control rules in de facto open access, Territorial user rights for fishers or TURFs). Most small-scale fishers in Chile belong to a fishing association as membership allows for collective bargaining, and it is a legal requirement to apply for TURFs over benthic resources (known locally as Management and Exploitation Areas of Benthic Resources or MEABR)^24^ (see the “Methodology” section for more details on the study site).

We ask how do people within fishing communities trust, cooperate and bargain in a game setting and how do socioeconomic characteristics (specifically age, income, gender, number of household members, marital status, being the household’s income provider, and having a secondary source of income besides fishing) influence this behavior? The games used an unframed setting with double blinds (participants did not know who they were playing with) in which pairs/groups were randomized—roles were fixed but partners were not. Aiming to accurately represent gender balances in Chilean small-scale fisheries, we attempted to maintain a representative proportion of women and men without restricting participation (for more details see “Methodology” section).

## Results

A total of 192 subjects participated in the study, and 1/3 were women (self-reporting). Participants’ average characteristics (socioeconomic variables) are similar across games with the exception of ‘household income provider’ (Table S1 in Supplementary Materials). Total average earning across games was 10.4 USD for the trust game, 8.7 USD for the bargaining game, and 13.8 USD for the public goods game (the minimum wage in Chile was ~ 2.36 USD per hour at the time of the study).

### Public goods game

Public goods game had participants, in groups of three, decide how much from an endowment they wanted to contribute to a group fund that doubled the sum of contributions and split them equally among all members^25^. Contributions to the public good were, on average, 5.46 USD (66% of their endowment). Broadly, respondents tended to cooperate; only ~ 4% of participants engaged in freeriding (contributing zero) (Figure S1 in Supplementary Materials). We found that gender affected contributions. Women contributed, on average, 6.90 USD (SE 0.40) and men 4.77 USD (SE 0.48); this difference is statistically significant (p-value 0.01 using Mann–Whitney–Wilcoxon test). Effect size tests (Cohen’s D) indicates a medium difference in contribution between men and women (d = − 0.77). An important number of participants (41%) contributed all their endowment to the common fund (15 men, 14 women), but only male participants (16% of them) contributed less than 20% of their endowment (Figure S1 in Supplementary Materials). No other differences within discrete characteristics were observed using Mann–Whitney–Wilcoxon test (Table S2 in Supplementary Materials).

**Figure 1:**
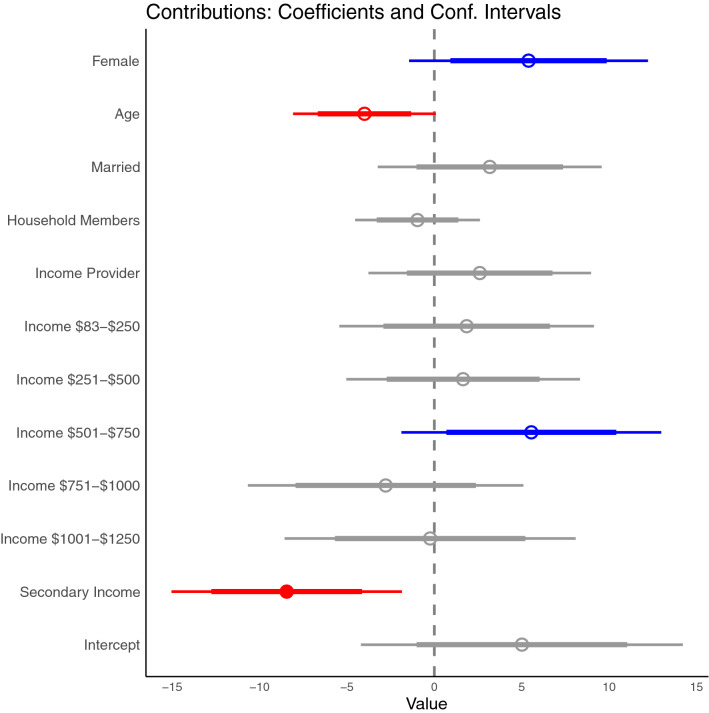
Coefficient plot of how contributions are related to gender, age, marital status, number of household members, whether a subject was the income provider, the levels of monthly income (in USD), and whether the subject had a secondary non-fishing income. Thick lines represent 80% confidence interval, while thin lines are 95% confidence intervals.

The Bayesian truncated model of contributions corroborates descriptive findings regarding gender and indicates a weak relationship to contributions, women contributed more than men 88% of the time (post-hoc comparisons). Controlling for the influence of other variables, the model also shows a negative relationship with contributions for age and having a secondary income. There were no other significant differences in other individual socioeconomic variables. (Fig. 1 and Figure S2 in Supplementary Materials).

### Trust game

**Figure 2:**
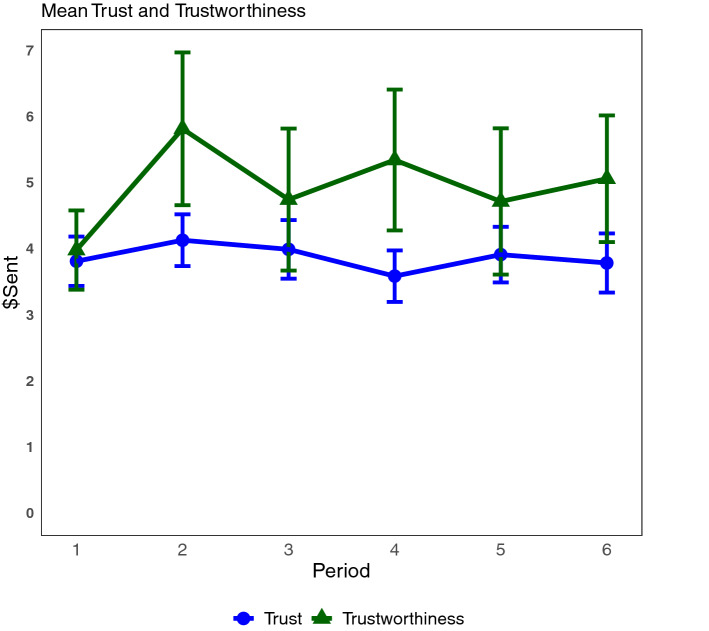
Average amount sent (Trust) by senders and average amount sent back (Trustworthiness) by receivers per round. Vertical lines represent standard errors. Endowment for buyers/sellers was $6.67, average amount received by receivers (after being multiplied) was $11.59.

The trust game was a two-stage game in which a sender could send money to a receiver (sender and receiver were endowed with an equal amount of money). Before reaching the receiver the money was tripled, then the receiver had to decide if he/she wanted to send anything back^26^. Trust (money sent by senders) and trustworthiness (money sent back to senders from receivers) was on average 58% (of the endowment) and 47% (of the money received), respectively, and stayed relatively constant throughout the rounds (Fig. 2). Senders sent positive amounts 93% of the time. Reciprocity after receiving a positive amount occurred 80% of the time; while in six occasions receivers who were sent nothing sent money back from their endowment (Figure S3 in Supplementary Materials).

We found that respondent’s income role (i.e., being an income provider) and income sources (i.e., having a secondary income) affected trust and reciprocity behaviours in different ways. Income providers sent (senders) 22%, and sent back (receivers) 29%, more than people that were not the income provider of their household (W = 2037 for senders and W = 1495 for receivers, p-values < 0.01 using Mann–Whitney–Wilcoxon test). In contrast, senders with a secondary income sent 16% more, while receivers with a secondary income sent back 34% less, than those who had income only from fishing (W = 3658 and p-value 0.04 for senders and W = 2854 and p-value 0.07 for receivers, using Mann–Whitney–Wilcoxon test) (Table S2 in Supplementary Materials).

We also found gender differences in trust and reciprocity. Women trusted and reciprocated more (28% and 64% in average respectively) than men (W = 2054 and p-value < 0.01 for trust and W = 1340 and p-value < 0.01 for reciprocity, using Mann–Whitney–Wilcoxon test). Men sent on average 56% of their endowment while women sent 71% (Table 1). Receivers who were men returned an average of 43% of the money they received, compared to an average of 65% returned by receivers who were women; this translated in an average return on trust (for senders) of 29% when the receiver was male and 96% when female. Effect size tests (Cohen’s D) between genders indicates medium differences for trust and reciprocity (d = − 0.49 and − 0.53 respectively).

**Table 1:** Trust and trustworthiness by rounds and gender.

	Trust				Trustworthiness			
Gender	Male		Female		Male		Female	
Round	Mean	SE	Mean	SE	Mean	SE	Mean	SE
1	3.84	0.48	4.19	0.68	3.41	0.72	6.33	0.57
2	4.42	0.44	4.63	0.71	5.22	1.40	8.50	2.02
3	3.87	0.61	4.81	0.66	4.49	1.44	6.19	0.25
4	3.33	0.45	5.00	0.61	4.55	1.28	8.57	1.68
5	3.26	0.55	5.30	0.55	4.19	1.35	7.02	1.94
6	3.60	0.58	4.66	0.86	4.63	1.24	6.88	1.06

*SE* standard errors.

**Figure 3:**
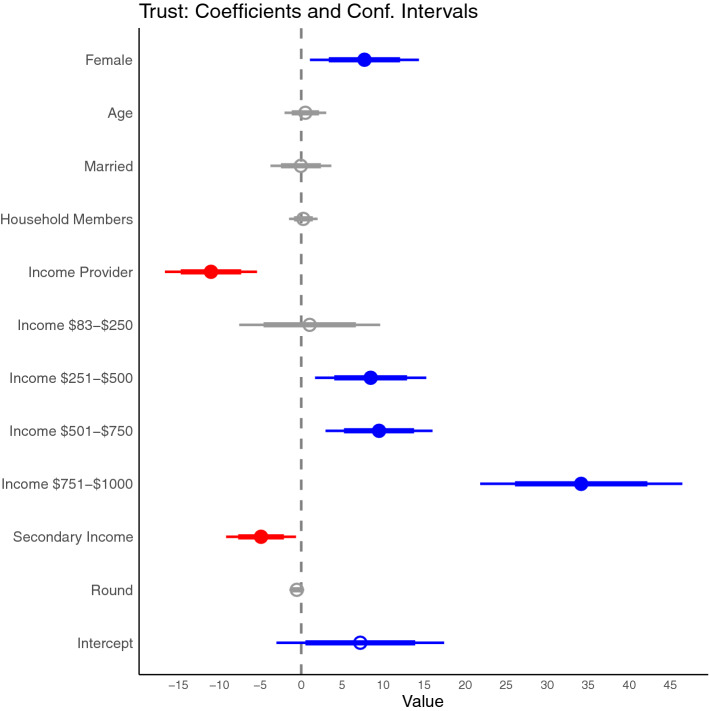
Coefficient plot of how the amount sent by senders (Trust) is related to gender, age, marital status, number of household members, whether a subject was the income provider, the levels of monthly income, whether the subject had a secondary non-fishing income, and the round number. Thick lines represent 80% confidence interval, while thin lines are 95% confidence intervals.

**Figure 4:**
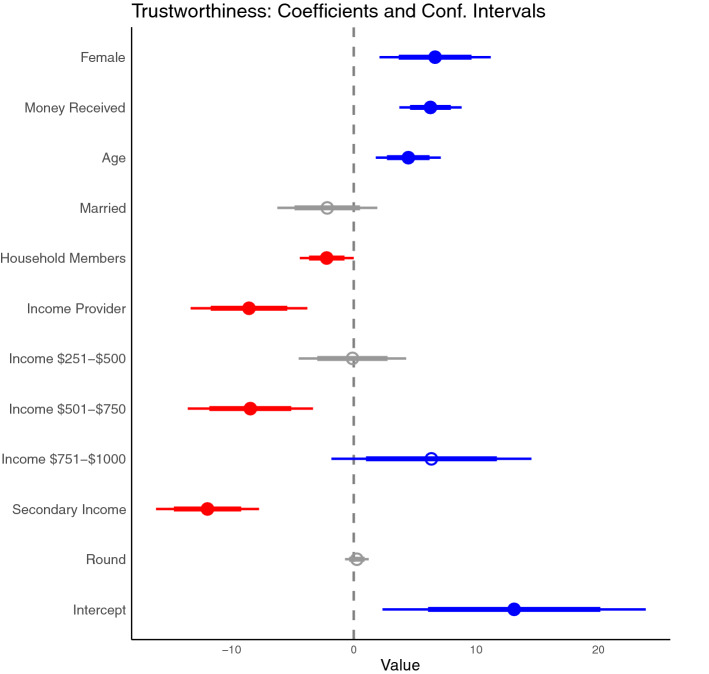
Coefficient plot of how the amount sent by receivers (Trustworthiness) is related to gender, money received (i.e., money sent by senders tripled), age, marital status, number of household members, whether a subject was the income provider, the levels of monthly income (in USD), whether the subject had a secondary non-fishing income, and the round number. Thick lines represent 80% confidence interval, while thin lines are 95% confidence intervals.

These findings of gender and income differences are supported by the Bayesian truncated models for trust and reciprocity. Tukey adjust pairwise comparisons between factors indicate women have a positive influence in the amount sent by senders (trust) and the amount send back to senders by receivers (trustworthiness) when compared to men. In addition, being the household income provider and having a secondary income had a negative influence in trust and trustworthiness. The level of income suggests a positive relation to trust but negative to trustworthiness, although both influences are non-linear as income increases, compared to the lowest level of income. The number of household members and marital status does not seem to have a significant influence in either trust or trustworthiness while age is only positively related to trustworthiness. The amount of money received by receivers has a positive influence in the amount of money sent back to senders (Figs. 3, 4 and Figure S2 in Supplementary Materials).

### Bargaining game

**Figure 5:**
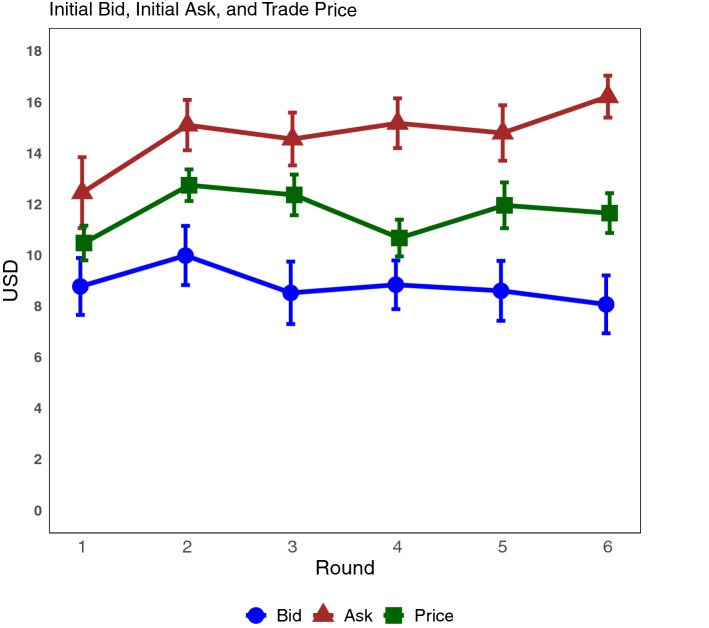
Average initial Bid, initial ask, and trade price per round. Vertical lines indicate standard errors.

The bargaining game had a buyer and a seller engaging in bilateral trading for a hypothetical box in which sellers were endowed with the box and buyers with money. In order to make money they had to reach an agreement on a price; sellers getting the price and buyers getting their endowment minus the price^27^. Agreed prices in the bargaining game were on average 16.5% higher than the equitable distribution (10 USD), resulting in sellers earning 39.4% more than buyers. The overall average equilibrium price (11.65 USD) in the bargaining game is slightly above the expected equitable distribution in the first round and stays over it throughout the rounds (Fig. 5). Most trades (86%) were concluded by the fourth offer and only 9% of transactions were not concluded. Initial offers from buyers (bids) we closer to the equitable distribution than sellers’ (asks) throughout the rounds drifting apart towards the last rounds (Fig. 5); the initial offer was, in average, 14.79 USD for sellers and 8.80 USD for buyers.

We found few and weak gender differences in the bargaining game and these were connected to the role of participants. Initial bids and asks from women were similar to those from men for the first three rounds but drifted apart in the last three; with women increasing asks and reducing bids when compared to men (Table 2). Effect size tests (Cohen’s D) indicates small differences in earnings from bargaining between genders for buyers and sellers (d = − 0.27 and 0.22 respectively). Women buyers earned, on average, ~ 19% more than men buyers (9.46 USD and 7.97 USD respectively), and ~ 10% less than men sellers (10.74 USD and 11.97 USD respectively); but these differences were not statistically significant (W = 2446 and p-value 0.11 for buyers and W = 2310 and p-value = 0.25 for sellers, using Mann–Whitney–Wilcoxon test). Finally, only ~ 19% of transactions not concluded involved women. We also found that marital status intersected with respondent’s role in the game. Married buyers earned 35% less than unmarried buyers (W = 1820 and p-value < 0.01 using Mann–Whitney–Wilcoxon test). Effect size tests (Cohen’s D) indicates medium differences in earnings from bargaining between married and unmarried buyers (d = − 0.63). Other socioeconomic variables exhibit no significant differences regardless of the role of respondents (Table S2 in Supplementary Materials).

**Table 2:** Equilibrium price by rounds and gender.

Gender	Male		Female	
Round	Mean	SE	Mean	SE
1	11.65	0.81	8.30	1.30
2	13.12	0.71	11.81	1.27
3	13.38	0.89	10.17	1.54
4	10.41	0.95	10.70	1.28
5	11.85	1.07	11.18	1.88
6	11.51	0.99	11.68	1.38

*SE* standard errors.

**Figure 6:**
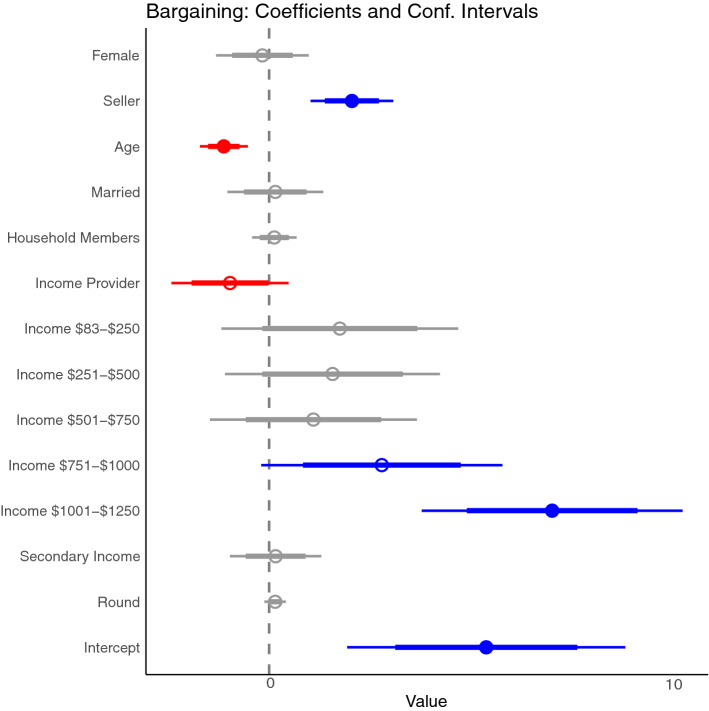
Coefficient plot of how the earnings from bargaining are related to gender, their role (buyer/seller), age, marital status, number of household members, whether a subject was the income provider, the levels of monthly income (in USD), whether the subject had a secondary non-fishing related income, and the round number. Thick lines represent 80% confidence interval, while thin lines are 95% confidence intervals.

Accounting for role and all characteristics, the Bayesian model for bargaining prowess (earnings from bargaining) suggests the role (buyer/seller) of the participant has a strong influence on earnings, while gender has no influence on its own. Being a seller has a positive influence on bargaining earnings compared to being a buyer. The model also suggest age of the subject has a negative relationship to earnings while higher levels of income seem to increase bargaining earnings (this relationship is not linear); being the household income provider has a weak negative relationship (82% of the time) with earning. All other variables do not seem to influence earnings from bargaining when accounting for role. (Fig. 6 and Figure S2 in Supplementary Materials).

## Discussion

The use of three distinct games to study cooperation, trust, and bargaining in fishing communities requires a brief discussion of each of them before turning to the discussion of overall implications of games in the field.

For the public goods games, cooperation was high—compared to previous lab experiments^25,28^—with low levels of freeriding and a significant percentage (41%) of participants contributing all their endowment. These results could be influenced by our settings (one-shot, small group sizes, large expected benefit from unit contribution)^25,29^, or reflect the context of the communities, suggesting high levels of cohesion and cooperation^30^. Indeed, our studies were conducted in fisheries associations (syndicates). Contributions were influenced by gender and age. Contrasting results from previous studies, we found women to contribute more than men^31^, and older people to contribute less than young people^12,32^. Income has a less clear influence in contributions, while personal income and being the household income provider do not seem to affect contributions, having a secondary non-fishing income seems to decrease them^11^. It could be expected that having a secondary income produces a higher total income, but we did not find evidence of this in our data. Therefore, we speculate that participants with a secondary income are less financially dependent on fishing and may be more socially distant (i.e., less available to engage in social activities and share experiences) to others in the fishing community. We expect people less dependent on the fishery to be less willing to contribute to the success of it (e.g., enforce rules), and be less willing to take risks associated with the fishery^33^. Overall, evidence for distinct differences in contributions to public good games (by either gender, age, income, or other) are still inconclusive in the literature, suggesting high dependency on cultural context and game settings^31^. For instance, women’s higher cooperation in our study likely reflects socio-cultural aspects about gender roles in Chile, and in these small-scale fishing communities in particular.

In the trust game we observe high levels of trust (58% of endowment) and trustworthiness (47% of the money received) when our settings (equal endowment to both roles, double blind, non-student participants, and random payment) are taken into account^31,34^, but these levels are not different compared to other games conducted on the field^20,35^. Gender seems to be a strong predictor of trust and trustworthiness in Chile. The role of gender on trust and trustworthiness has been mixed in the past^36–39^; this ambiguity is sometimes attributed to trust games confounding trust and risk—as sending money to another person involves uncertainty of returns^40–42^. Confounding trust and risk has been used to suggest women trust less than men since, in certain contexts, women can be more risk averse than men^31^, and ‘trust’ less when asked to send money with an uncertain return. We do not find evidence to support the previous hypothesis as women trust and are more trustworthy than men in our study. Income seems to play a weak role in trust and trustworthiness^43^. Being the household income provider and having a secondary income have a negative relationship to both trust and trustworthiness, but personal income has a positive—although not linear—relationship with trust, while its relationship to trustworthiness is inconsistent and ambiguous. The relationship of income and trust may be associated with risk tolerance, as higher levels of income can make people more willing to send money in hopes of higher returns^40–42^. At the same time, greater household responsibility (income provider and higher number of household members), and being less dependent (secondary non-fishing income) on fishing may mean participants are less willing to send and return money. The levels of trust and trustworthiness we found could also be due to a small social distance between the participants—participants do not know who they are playing with, but they do know it is someone from the community^44^.

In the bargaining game we found equilibrium prices to be above the equal distribution, resulting in higher profits for sellers. Endowing seller with a hypothetical object could have resulted in higher initial prices (asks), that were then used as reference by buyers^45^. The difference could also reflect higher bargaining power from sellers due to their day-to-day interactions with formal markets and set prices. In places where markets are rudimentary and bartering is the norm, the opposite relationship (bargaining power from buyers) can be found^20^. Bargaining prowess is strongly dependent on the role (buyer/seller) of the participant but not on particular socioeconomic characteristics. Income had a weak positive influence over earnings, while age and being the household income provider has a weak negative influence. The absence of evidence regarding the relationship between gender and bargaining should be noted. While several studies have suggested women’s outcomes from negotiations are worse than men (often from dictator and ultimatum games)^6,7,31^, we did not find evidence earnings from bargaining are lower for women than men when the role of the participant (buyer/seller) and other socioeconomic variables are taken into account. The only gender difference we see is in unsuccessful bargaining, with the majority not involving women, suggesting men might be less willing to compromise or having a negotiation strategy less flexible than women^46^.

Overall, our study strengthens the argument that results from experiments carried out in the field are nuanced and context specific, perhaps highlighting aspects which should be considered when addressing external validity of experimental settings, and economic games in particular. Since the formalization of experimental economics^23^, the issue of external validity of games (i.e., applicability to the real world), and their implications across cultures has continued to rise in importance^47,48^. The artificiality of economic games carried out with students has created concerns regarding generalization of their results. The variability of results across studies using public goods games^25,28^ and trust games^34^ has also contributed to concerns of external validity and context dependency in economic games in general^49,50^. Recent studies suggest the direction (instead of the magnitude) of the behavioral differences observed in games may be more readily generalized^51,52^. The measure of external validity also raises questions on both sides of the argument, as parallel situations between the lab and the field are not always obvious. Generalization across cultures presents a higher hurdle for the external validity of games, suggesting studies should be carried in the field, taking into account context and culture. We do not test the external validity of our results explicitly, but our results are consistent with previous research using common-pool resource games, and encompassing some of the same fishing communities, that showed pro-social behavior was linked with well-functioning fishing association^18,30^.

Our findings reinforce the importance of understanding pro-social and bargaining behavior of people who are likely to engage in such behaviors in day-to-day life. Further empirical investigations in the field will be important for understanding behaviors that are relevant for governing natural resources, especially common-pool or co-managed resources like fisheries. Our findings suggest that for fishing communities, individual characteristics can play an important role when looking at pro-social behavior, bargaining power, and its determinants. This study suggests there is a high willingness to engage in pro-social behavior in these Chilean fisheries, which shows potential for effective collaborative management of the commons, or indeed reflect already existing cooperative behavior^30^. In addition, this study shows fishers have bargaining power as sellers, which suggest they have the potential to obtain better (or fairer) prices when negotiating catch sales. This willingness to engage in pro-social behaviors and bargaining may be enhanced or limited by the actual context, such as the institutions that govern the commons, power dynamics, or the social identities of fishers within the community. As participants in our study were all members of fishing associations, our findings do not allow us to identify if pro-social behavior is intrinsic to people in fishing communities, if it is a result from membership in fishing associations, or if it is a self-selection mechanism in which pro-social people are more willing to be part of a fishing association. Clarifying the underlying mechanisms should be the focus of future studies.

Here we show that the likelihood of behaving pro-socially and the presence of bargaining power can be associated to socioeconomic factors. While there is growing attention to the role of gender shaping access and benefits from fisheries^53,54^, our findings emphasize that multiple socioeconomic characteristics shape behaviors and not all of them do it in an intuitive way. For the participants in our study, being married had no influence on cooperation, trust, and bargaining gains; while being part of a large household had marginal influence only in trustworthiness (decreasing it). Older people were less cooperative but more trustworthy and willing to compromise when bargaining. Income had a weak influence in all behaviors; higher income earners were more willing to cooperate and trust, and did better when bargaining, but were less trustworthy. Women were more pro-social and did as well as men when bargaining. Household income providers trusted less and were less trustworthy, but were more willing to compromise when bargaining and cooperated the same as people who were not income providers. People with secondary non-fishing income manifested less pro-social behaviors, but when bargaining did as well as people whose income depended only of fishing.

Our study is not exhaustive in the types of variables that may influence pro-social behavior. Other characteristics (such as education, religious beliefs, political affiliation, and wealth) may also have an effect on cooperation, trust, trustworthiness, and bargaining prowess; particularly in other communities and different contexts, and warrant further study. In addition, it is possible that the interaction between characteristics (e.g., between gender and age), would also shape behavior^9^. Due to data limitation, our analysis only accounts for the influence different variables may have on pro-social behavior and bargaining, but does not look at the interrelationship between them. The role of intersectionality between characteristics should be taken into account when looking at pro-social and bargaining behavior in future studies.

Our findings, and their contrast with other studies, highlight that rather than intrinsic differences between people determining pro-social or bargaining behavior, context, culture, and experimental design can affect results^28,31,34^. For gender in particular, we found strong evidence for differences in trust and trustworthiness behaviors among genders, moderate evidence for differences among genders in cooperative behaviors, and no evidence for differences in bargaining among genders. Thus, findings on gender differences in behavioral games need to be interpreted within a broader, theoretically informed lens that challenges (rather than reinforces) stereotypes^55^. Our findings add weight to the need to understand context-specific behaviors and underscore the utility of expanding experimental games to field settings.

## Methodology

### Research site

The study was conducted in a 30 km stretch in the coastal area of the San Antonio province in Chile. The area encompasses a few small-scale fishing communities and local fishing associations (unions or syndicates) with members of similar characteristics (Table S1 in Supplementary Materials). Three fishing associations agreed to participate in the study, each one from a different community (San Antonio, El Quisco, and Las Cruces). The size of the fishing communities in the study ranged from 40 to 100 members and their main activity is commercial fishing for the national market (e.g., hake ‘*Merluccius gayi gayi’*, squid ‘*Dosidicus gigas’*, Gastropods ‘Concholepas concholepas’, and kelp ‘*Lessonia sp.‘*) Chile relies on a combination of open access and territorial use rights (TURFs) to manage marine resources extraction, with fishing associations playing an important role in resource management and conservation^24,56^. The establishment and consolidation of fishing associations in Chile resulted from the formalization of stablished social networks in fishing communities incentivized by the Fishing and Aquaculture Law of 1991 (modified and renamed Fisheries Law in 2013). Among others, the law granted fishing associations the ability to request exclusive territorial user rights over areas for management of benthic resources; representation in establishing—and distributing—national and regional fishing quotas; and access to technological, scientific, and financial support for resource management^24,57,58^. Belonging to a fishing association can also provide secondary benefits such as collective bargaining and risk sharing, but it can also constrain fishers’ freedoms (e.g., fishing methods, extracted species, and quantity extracted). One of the main objectives of the fishing law incentivizing the formal designation of fishing association was resource sustainability; however, the evidence suggests short-term economic incentives might be stronger than long-term sustainability for some Chilean fisheries^59–61^.

### Subjects

The research was approved by the Human Research Ethics Committee (HREC) of James Cook University. All experimental procedures in this study were carried in accordance with the guidelines and regulations approved by the HREC for social studies involving human subjects. Informed consent was obtained from all subjects before they participated in the study. Only adults (18 years old or older) were allowed to participate in the study, with subjects self-selecting randomly to participate in each of the independent games. Careful attention was paid to maintain gender representativeness (70/30 men to women ratio) through the games by conducting experimental sessions in times of low fishing activity.

### Experimental design

Our experimental design consisted of three games: a trust games, a bargaining game, and a public goods game. All games were played independently of one another; the bargaining and trust games were played six rounds (plus a practice round excluded from the analysis) while the public goods game was played only once (one-shot).

### Setting

Standard trust^26^, bargaining/trade^27^, and public goods games^62^ were programed using the computer software z-tree^63^. The sessions were conducted in three different location across the research site to facilitate attendance. We conducted 16 sessions (five of trust, five of bargaining, and six of public goods) with 12 subjects in each session. To make their decisions subjects had to input the amount of money (in CLP) they wanted to send, ask/offer, or contribute (depending on the game) using a numeric keyboard and a touch screen computer. For all games, instructions were read out loud before starting. Instructions were also given on paper and shown on the computer screen to facilitate understanding; communication was not allowed between subjects and questions were addressed individually. At the end of the games, subjects were asked to self-report gender (male, female, other), age, household monthly income (in brackets), number of household members, marital status, if they were the major providers of income in the household, and whether they had another source of income besides fishing.

### Public goods game

For the public goods game subjects were randomly assigned to groups of three and received an equal endowment of 8.3 USD (5000 CLP). From their endowment subjects had to decide how much they wanted to contribute to a group fund. Individual contributions in a group were then added, doubled, and divided equally (marginal per capita return of 0.66). To contribute, subjects had to input the desired amount and confirm it by selecting submit. All decisions were simultaneous and independent. Information on the computer screen included the endowment, and their contributions and earnings after all decisions were made.

### Trust game

For the trust game subjects were randomly paired and assigned a role (sender/receiver). Each round the pairs were reshuffled but the subjects’ roles maintained. Both subjects (roles) in a pair were endowed with 6.67 USD (4000 CLP) each. Each round senders had to decide how much, if any, of their endowment they wanted to send to the recipient, before reaching the recipient the money sent was tripled by the computer. Similarly, after receiving the money from the senders, receivers decided how much, if any, of their money (received plus endowment) they wanted to send back to the sender. Decisions were made sequentially and receivers had no obligation to reciprocate. To make their decisions subjects had to input the amount using the numerical keyboard and touch the screen to confirm their decision (any amount up to the total amount of money they had could be sent to their counterpart, including the extremes). Information on the computer screen included the subjects’ role, the round they were playing, the money they had, the money they received, and how much they made after all decisions.

### Bargaining/trade game

Similar to the trust game, at the beginning of the bargaining game subjects were randomly assigned a role (buyer or seller) and then randomly paired each round. Each pair consisted of one buyer and one seller and roles were maintained for the entirety of the game. Sellers, endowed with a hypothetical resource box, had to sell the goods to their counterpart, while buyers, endowed with 20 USD (12,000 CLP), had to buy it. Both buyer and seller were asked to submit offers (bids/asks) to exchange a box, the range of the bids/asks was from zero to 20 USD. The moment a subject submitted an offer (by typing the offer and selecting submit on the screen) it was displayed in their counterpart’s screen. After receiving an offer, subjects could decide to accept the offer (agreeing on a price) or submit a counteroffer. Subjects had a time limit of 3 min to agree on a price for the transaction. An initial offer by both buyer and seller was not needed to conclude a transaction, as long as one of them submitted an offer, the other player could accept it and end the transaction. Although making an offer was not mandatory, if no price was agreed by the end of the 3 min (no transaction), both players earned zero in that period (subjects were informed of this feature before and during the game—a blinking timer in the screen indicated the time left in each period). A player could submit a new offer at any point during the 3 min, even if he/she did not receive a counteroffer, but only the latest offer could be accepted. After conducting a transaction, sellers earned the price agreed and buyers earned their endowment minus the price. Information on the computer screen included the subjects’ role, the round they were playing, last offer made, counterpart’s offer (if any), and their earnings after all decisions were made (zero if no price was agreed).

### Payment

Subjects received individual payment (cash) in private at the end for each of the games. For multiple rounds games (trust and bargaining), only one of the rounds was selected at random and paid. All payments were made using local currency (Chilean Peso, CLP. 1 USD $\cong \hspace{0.17em}$600 CLP in January 2018 exchange rate).

### Analysis

The analysis focusses on the decisions, or the result from the decisions, made in the different games and their relationship (or lack of) to the gender of the subjects. Trust is analyzed by looking at ‘money sent’ (from senders), reciprocity by ‘money sent back’ (from receivers), bargaining by ‘earnings’, and cooperation by ‘contributions’ (to the group fund). In this study trust, reciprocity, bargaining, and cooperation are examined with Bayesian mixed effect models using the Hamiltonian Monte Carlo algorithm implemented in Stan through the brms package^64^ in the statistical software R (v 4.0.3). The models attempt to explain the relationship that trust, reciprocity, bargaining, and cooperation might have to gender—while accounting for the influence of socioeconomic factors and other controls. With the exception of bargaining, all models incorporate a gaussian truncated distribution of the independent variable. Trust and cooperation were truncated at zero (lower bound) and at the endowment (upper bound); reciprocity was truncated at zero (lower bound) and endowment times four (upper bound)—this is, endowment plus the maximum possible money received (three times the endowment of the sender) (Figure S4 in Supplementary Materials). The simulations used four chains of 10,000 iterations with 7000 burn in, leaving 12,000 samples in the posterior distribution of each parameter. Priors were assumed to follow a normal distribution with a mean of zero (weakly informative priors) for all the coefficients in all models. The distributions of the different chains in the different models as well as the scale reduction factor (R-hat ~ 1) indicated convergence and stability in the models; however, the models are not able to fully replicate some focal points (due to denomination bias) in the data (Figure S5 in Supplementary Materials). A random effect was included to account for potential intrinsic differences in the (three) different areas (communities) where the games took place. All models also included the self-reported covariates age, marital status, number of household members, household income provider, income (in brackets), and existence of a secondary income not related to fishing (see Table S1 in Supplementary Materials). Other covariates included were: round in the models for trust, reciprocity, and bargaining (to account for learning behavior within the game); money received (from senders) by receivers in the model for reciprocity; and role in the model for bargaining (to account for framing). No multicollinearity was observed between the covariates. Covariates age, household members, and money received were standardized (mean centered and divided by standard deviation) to account for scale problems. Residuals were normally distributed for bargaining and with a slight indications of fat tails; which can be expected in truncated models (Figure S6 in Supplementary Materials). Models without truncation did not provide different qualitative results, and while measures of cross validation (LOOIC and K-fold) tend to improve slightly, the relevance of observations at truncation points is omitted and estimate distributions become bias towards zero (with models becoming less flexible). Post-hoc pairwise comparisons were done using Tukey tests^65^ implemented with the emmeans package in R. To complement the information from the Bayesian analysis we use descriptive statistics, effect size (Cohen’s D), and non-parametric tests (Mann–Whitney–Wilcoxon) when appropriate.

## Supplementary Information

Supplementary Information.

## Data Availability

The data used in this study is available at 10.25903/60k1-ax48.
