# Supplementary material for: An experimental look at trust, bargaining, and public goods in fishing communities
Source: Sci Rep. 2021 Oct 21;11:20798. doi: 10.1038/s41598-021-00145-5 (PMC8531345; doi:10.1038/s41598-021-00145-5)
Supplement: Supplementary file 1 — Supplementary Information. [file 41598_2021_145_MOESM1_ESM.pdf]

Supplementary Materials for

An Experimental look at Trust, Bargaining, and Public Goods in Fishing Communities

Cristian A. Rojas*, Joshua Cinner, Jacqueline Lau, Cristina Ruano-Chamorro, Francisco J. Contreras-Drey, Stefan Gelcich.

*Corresponding author. Email: crojas2501@gmail.com

This PDF file includes:

Figs. S1 to S6
Table S1 to S2
Data

Fig. S1.

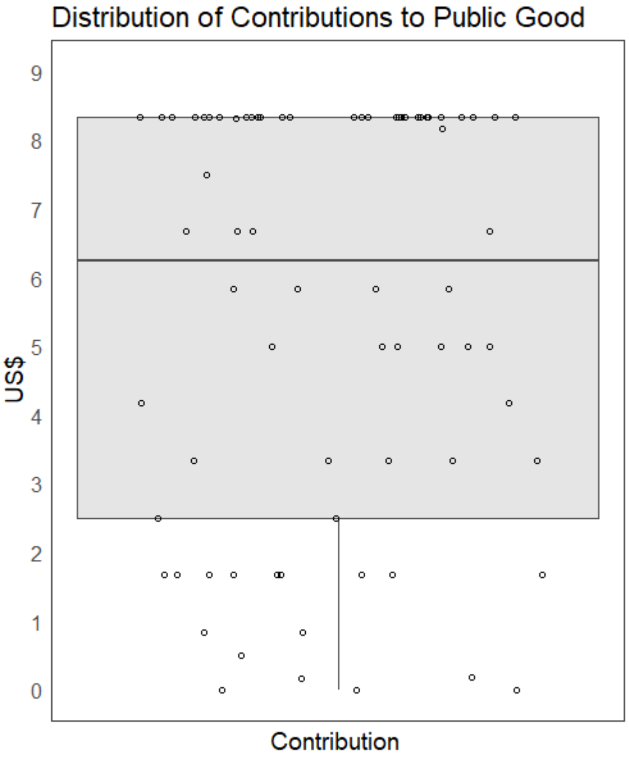

Figure S1: Distribution of contributions. Hollow circles represent data points (dispersion added for visual purpose). Endowment was ~$8.3

Fig. S2.

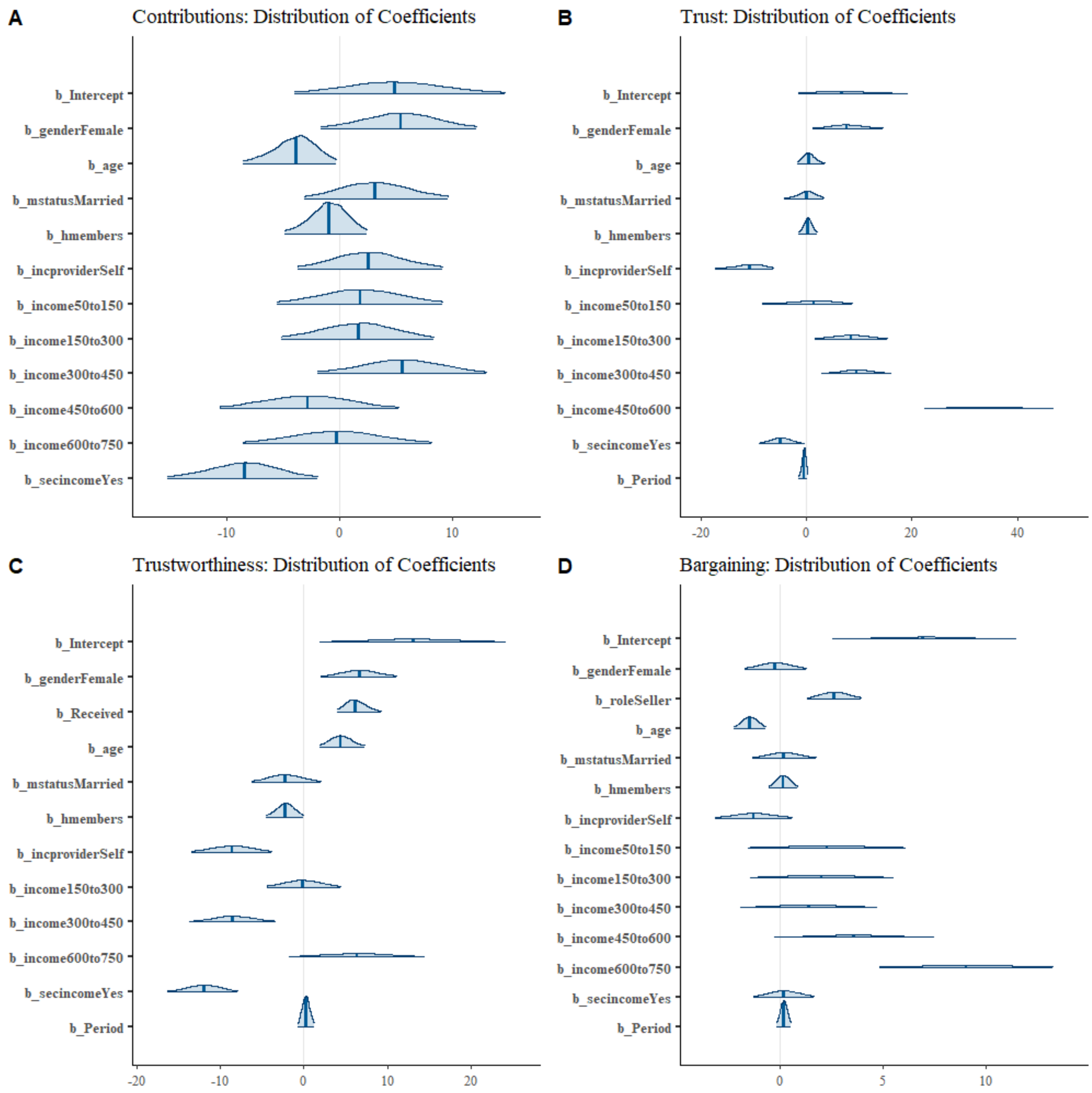

Figure S2: Posterior distributions of coefficients (median and 95% interval) from Bayesian models for: A) Contributions to Public goods; B) Trust (Money sent by senders in trust game); C) Trustworthiness (Money sent back by receivers in trust game); and D) Bargaining prowess (earnings from bilateral bargaining in Bargaining game)—not truncated, over socioeconomic and relevant variables (see Methods section for more details). Income levels' labels in thousand CLP.

Fig. S3.

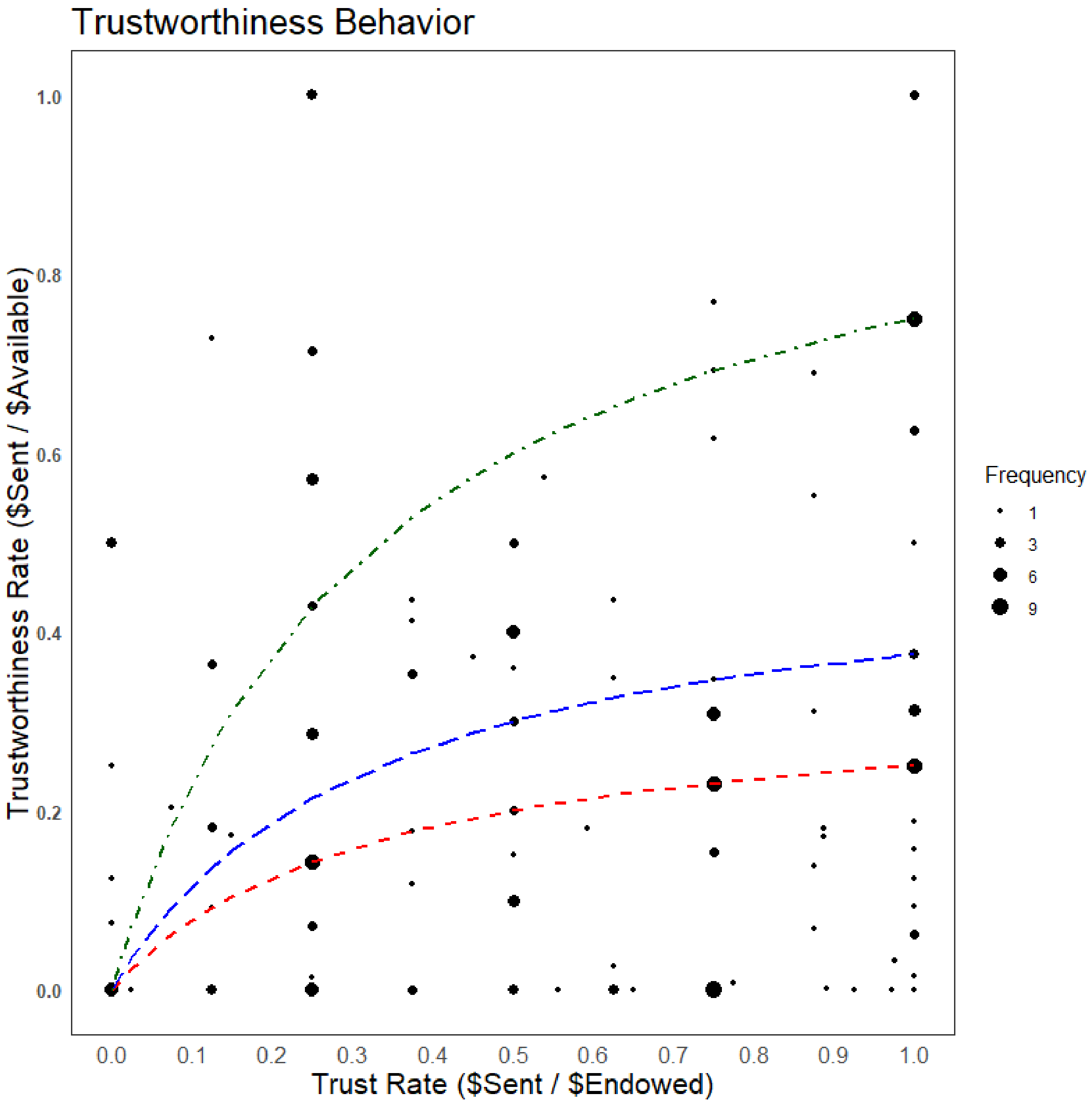

Figure S3: Relationship between rates of trust and reciprocity. The dashed line (red) indicate the threshold for recipients that returned the money originally sent to them (before being multiplied by three). The long dashed line (blue) indicates recipient that returned half (50%) of the money received (after being multiplied). The dot-dashed line (green) indicates recipients that returned all (100%) of the money received (after being multiplied).

Fig. S4.

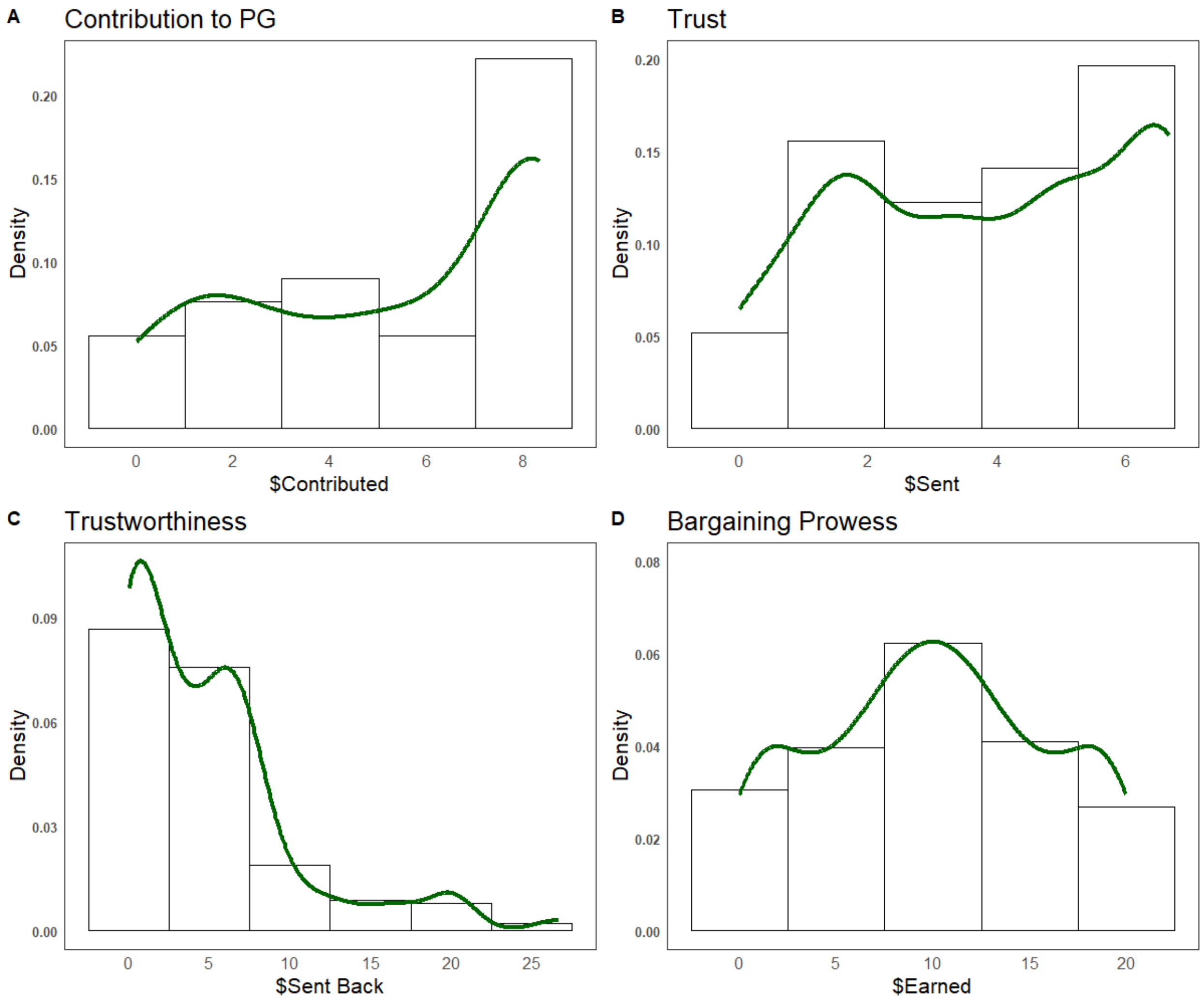

Figure S4: Histogram and density distribution for: A) Contributions to public goods, B) Trust (Money sent by senders in trust game), C) Trustworthiness (Money sent back by receivers in trust game), and D) Bargaining prowess (earnings from bilateral bargaining in Bargaining game).

Fig. S5.

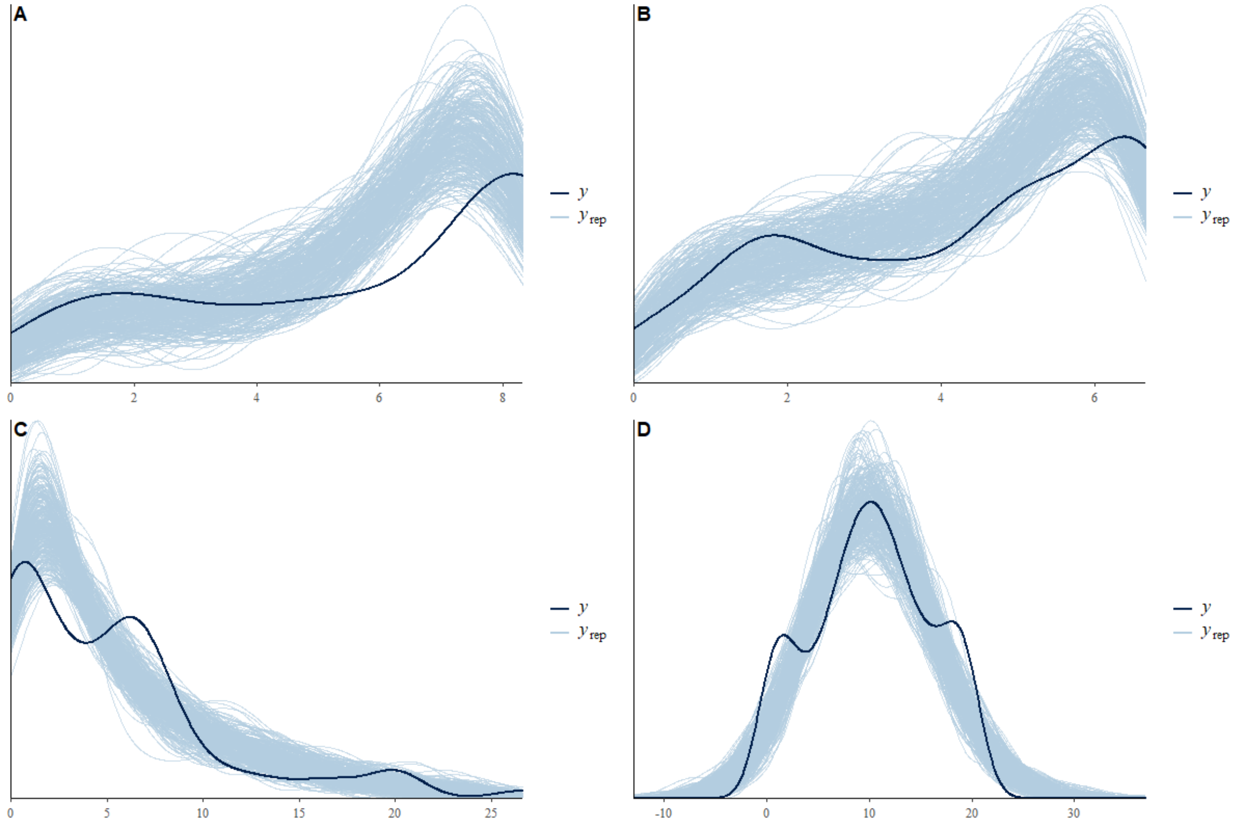

Figure S4: Distribution (y) and random sample of predicted distributions (y_{rep}) for: A) Contributions to public goods, B) Trust (Money sent by senders in trust game), C) Trustworthiness (Money sent back by receivers in trust game), and D) Bargaining prowess (earnings from bilateral bargaining in Bargaining game).

Fig. S6.

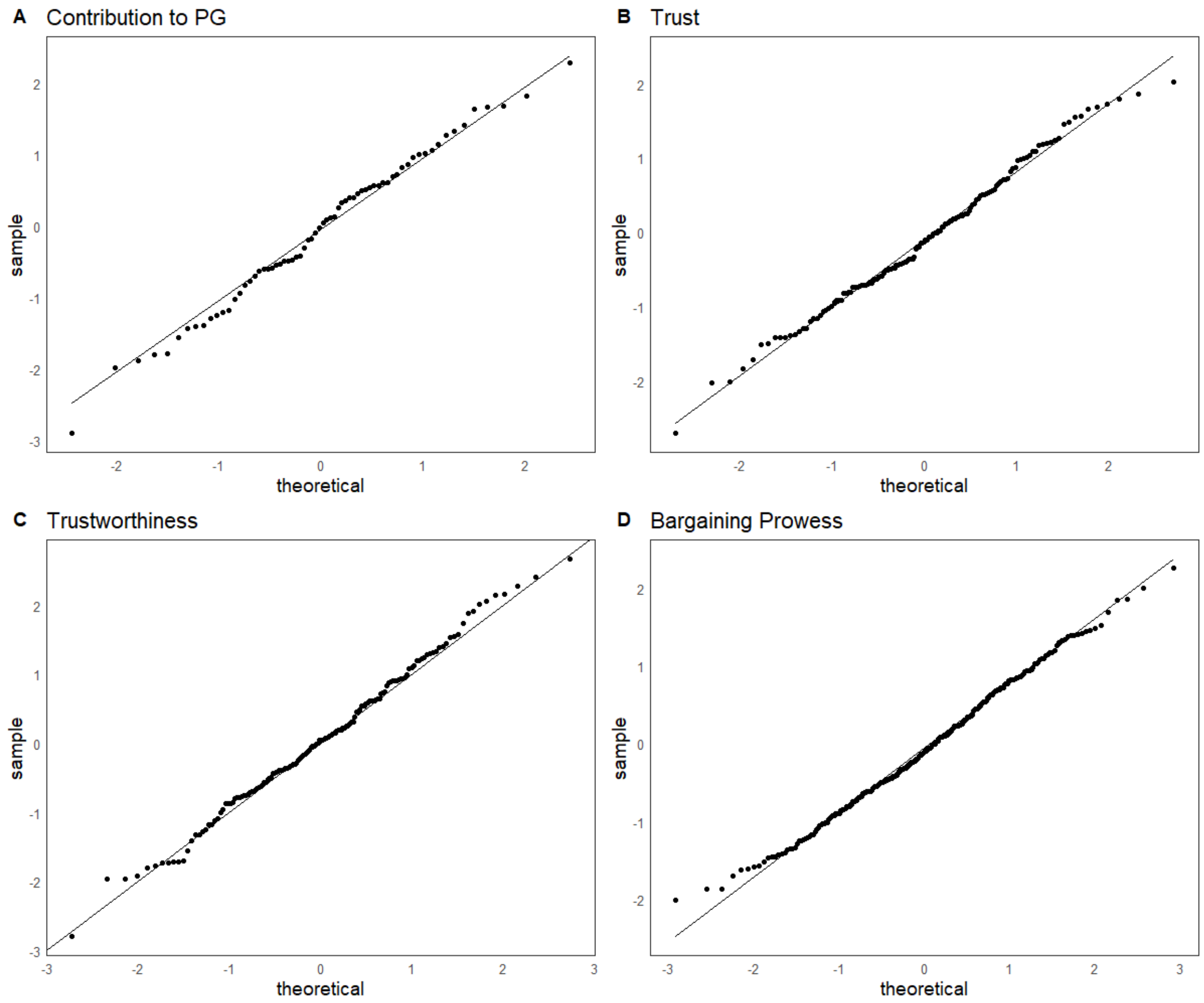

Figure S5: Quantile-quantile (QQ) plots of residuals from truncated Bayesian models for: A) Contributions to Public Goods, B) Trust (Money sent by senders in trust game), C) Trustworthiness (Money sent back by receivers in trust game), and D) Bargaining prowess (earnings from bilateral bargaining in Bargaining game)—not truncated.

Table S1.

Table S1: Summary of Socioeconomic variables and Earnings

Variable	Description	Game (Answer)	Mean / percentage	SD	N
Age	Age of the Subject	Trust	50.7	13.8	59
		Bargaining	47.8	14.6	57
		Public Goods	46.7	15.5	71
Income*	Monthly income in the household	Trust	$250-$500	-	51
		Bargaining	$250- $500	-	54
		Public Goods	$250-$500	-	70
Household Members	Number of household members	Trust	4.0	1.5	56
		Bargaining	3.6	1.4	56
		Public Goods	4.0	1.4	70
Married	Is the subject married?	Trust (Yes)	51%	-	57
		Bargaining (Yes)	45%	-	56
		Public Goods (Yes)	31%	-	70
Income Provider	Is the subject the household income provider?	Trust (Yes)	73%	-	56
		Bargaining (Yes)	80%	-	56
		Public Goods (Yes)	56%	-	70
Gender	Is the subject Male?	Trust (Yes)	71%	-	56
		Bargaining (Yes)	68%	-	56
		Public Goods (Yes)	61%	-	70
Secondary Income	Does the subject perceive income other than from fishing activities?	Trust (Yes)	52%	-	56
		Bargaining (Yes)	50%	-	56
		Public Goods (Yes)	46%	-	70
Earnings	Earnings in each of the games	Trust	$10.4	$6.4	60
		Bargaining†	$8.7	$6.4	60
		Public Goods	$13.8	$3.0	72

Note: Mean and standard deviations (SD) are expressed in USD (1 USD ≅ 600 CLP in January 2018). Income represents a range as it was measured in brackets. *For the level of income, the mode was used and converted to USD. †9% of total transactions did not reach an agreed price and resulted in zero earnings for buyers and sellers in a particular round.

Table S2.

Table S2: Summary of discreate variables by game.

Variable	Levels	Public Goods (Contribution)		Trust (Money sent)		Trust (Money sent back)		Bargaining* (Earnings)	
Marital Status	Married	$ 5.27	(0.69)	$ 3.91	(0.28)	$ 5.59	(0.57)	$ 9.41	(0.50)
	Not Married	$ 5.74	(0.41)	$ 3.99	(0.22)	$ 4.16	(0.54)	$ 10.51	(0.44)
Income Provider	Self	$ 5.36	(0.49)	$ 3.71	(0.21)	$ 4.71	(0.49)	$ 9.88	(0.36)
	Other	$ 5.88	(0.51)	$ 4.77	(0.29)	$ 6.60	(0.65)	$ 10.60	(0.81)
Gender	Male	$ 4.77	(0.48)	$ 3.72	(0.21)	$ 4.42	(0.51)	$ 10.28	(0.40)
	Female	$ 6.91	(0.40)	$ 4.76	(0.27)	$ 7.25	(0.56)	$ 9.90	(0.59)
Secondary Income	Yes	$ 5.30	(0.51)	$ 4.45	(0.30)	$ 4.35	(0.41)	$ 10.47	(0.49)
	No	$ 5.84	(0.49)	$ 3.84	(0.21)	$ 6.53	(0.91)	$ 9.82	(0.45)

Note: Values represent means, standard errors in parentheses. *Only including completed transactions.

Data.

The data can be found at: https://doi.org/10.25903/60k1-ax48 DOI: 10.25903/60k1-ax48
